# Thirty years of StAR gazing. Expanding the universe of the steroidogenic acute regulatory protein

**DOI:** 10.1530/JOE-24-0310

**Published:** 2025-02-06

**Authors:** Walter L Miller

**Affiliations:** Department of Pediatrics, Center for Reproductive Sciences, and Institute for Human Genetics, University of California, San Francisco, San Francisco, California, USA

**Keywords:** acute response, congenital lipoid adrenal hyperplasia, cholesterol, mitochondria, steroid

## Abstract

The current understanding of the biology, biochemistry and genetics of the steroidogenic acute regulatory protein (StAR) and its deficiency state (lipoid congenital adrenal hyperplasia, lipoid CAH) involves the complex interplay of four areas of study: the acute regulation of steroidogenesis, clinical phenomena in lipoid CAH, the enzymatic conversion of cholesterol to pregnenolone in steroidogenic mitochondria, and the cell biology of StAR. This review traces the origins of these areas of study, describes how they have been woven into an increasingly coherent fabric and tries to explore some remaining loose ends in this ongoing field of endocrine research. Extensive research from multiple laboratories has established that StAR is required for the rapid, abundant steroidal responses of the adrenals and gonads, but all steroidogenic cells, especially the placenta, also have StAR-independent steroidogenesis, whose basis remains under investigation. Lipoid CAH is the StAR knockout of nature whose complex (and unexpected) clinical features are explained by the ‘two-hit model’, in which StAR-dependent steroidogenesis and StAR-independent steroidogenesis are lost sequentially. StAR is targeted to mitochondria and acts on the outer mitochondrial membrane before being imported via the ‘translocase of outer membrane’ system and is then inactivated by mitochondrial proteases. A role for the ‘translocator protein’ (TSPO) has long been proposed, but an essential role for TSPO is excluded by recent transgenic mouse experiments. Crystal structures show that a StAR molecule can bind one cholesterol but does not explain how each StAR molecule triggers the import of hundreds of cholesterol molecules; this is the most pressing area for future research.

## Introduction

The key event in this story is the initial cloning of StAR from mouse Leydig MA-10 cells (Clark *et al.* 1994), which is being celebrated by the current series of papers about StAR in the *Journal of Endocrinology*. That landmark paper was the culmination of much prior work and was also the gateway to exciting, unanticipated areas of cell biology, genetics and medicine, as StAR is central to all aspects of cellular cholesterol trafficking and steroidogenesis. Detailed reviews have been published about the history of the discovery of StAR (Stocco & Clark 1996), regulation of StAR gene expression (Stocco *et al.* 2005), steroidogenesis and its disorders (Miller & Auchus 2011), intracellular cholesterol trafficking (Miller & Bose 2011, Miller 2017*a*) and the history of adrenal research (Miller & White 2023); these provide more detailed consideration and exhaustive literature citation than is possible here. This brief overview provides a timeline of events in four converging areas of study: mechanisms of the acute regulation of steroidogenesis, leading to StAR; lipoid congenital adrenal hyperplasia, the ‘StAR knockout of nature’; the chronic regulation of steroidogenesis at the level of the cholesterol side-chain cleavage enzyme, P450scc; and the ongoing studies about the cell biology of StAR. A timeline of some of the key events is shown in Table 1.

**Table 1:** Timeline of research concerning StAR.

1955 First autopsy reports of infant deaths with grossly enlarged, lipid-filled adrenals
1957 Defective steroidogenesis in these patients: ‘lipoid CAH’
1961 Conversion of cholesterol to pregnenolone requires 20α-OH and 22-OH intermediates
1963–68 ACTH’s action requires new adrenal protein synthesis
1966 Conversion of cholesterol to pregnenolone catalyzed by mitochondrial P450scc
1968 Cholesterol not converted to pregnenolone in lipoid CAH: ‘20,22-desmolase deficiency’
1980 Mitochondrial cholesterol import: the rate-limiting step in steroidogenesis
1983–1991 Orme-Johnson identifies 30-kDa ACTH-induced mitochondrial phosphoprotein in mouse adrenocortical cells and in LH-induced rat corpus luteum cells
1989–90 Reports that PBR translocates cholesterol from the OMM to the IMM
1991 Stocco confirms Orme-Johnson’s phosphoprotein in mouse MA-10 Leydig cells
1991 P450scc and candidates for cholesterol transfer proteins unaffected in lipoid CAH
1993 Gene for PBR normal in lipoid CAH
1994 Stocco *et al.* cloned cDNA for pp30; expression in MA-10 cells induced steroidogenesis without LH stimulation: StAR
1995 Lin *et al.* established the essential role of StAR: StAR mutations cause lipoid CAH and coexpression with the P450scc system in non-steroidogenic cells induced steroidogenesis
1995 Human StAR cDNA and gene cloned
1996 N-terminal deletions: StAR acts outside the mitochondria
1996 First large series of lipoid CAH patients, common mutations; two-hit model
1997 Confirmations of two-hit model by clinical and mouse studies
1997 Report that knockout of PBR in R2C Leydig cells inhibits steroidogenesis
1997 Phosphorylation of Ser195 needed for full StAR activity
1999 ‘START domains’ defined in plants and animals
1999 StAR acts as a partially unfolded molten globule
2000 N-238 MLN64 has StAR-like activity and an apparently similar structure
2000 Crystal structure of N-216 MLN64 suggests StAR acts in the IMM
2001 Only newly synthesized (extramitochondrial) StAR is active
2001 First report of P450scc deficiency
2002 StAR acts only on the OMM; activity based on OMM residency time
2005 PBR and StAR appear to cooperate
2005 Modeling of molten globule transition
2006 Non-classic lipoid CAH reported
2007 Cholesterol binding does not predict StAR activity
2007 StAR degraded by intra-mitochondrial Lon protease
2008 Molten globule of StARD6
2008 StAR activity requires contact with VDAC1 and PCP on the OMM
2009 Non-classic P450scc deficiency
2012 Sigma receptor links the MAM to the OMM
2014 PBR/TSPO knockout mice ruled out an essential role in steroidogenesis
2015 MAM regulates StAR/VDAC2 interaction
2017 ER chaperone GRP78 folds StAR in the MAM before delivery to the OMM
2023 Role of Tom40 in StAR import and activity

CAH, congenital adrenal hyperplasia; PBR, peripheral benzodiazepine receptor; OMM, outer mitochondrial membrane; IMM, inner mitochondrial membrane; MAM, mitochondria-associated membrane; TSPO, translocator protein.

## Lipoid CAH

**Figure 1:**
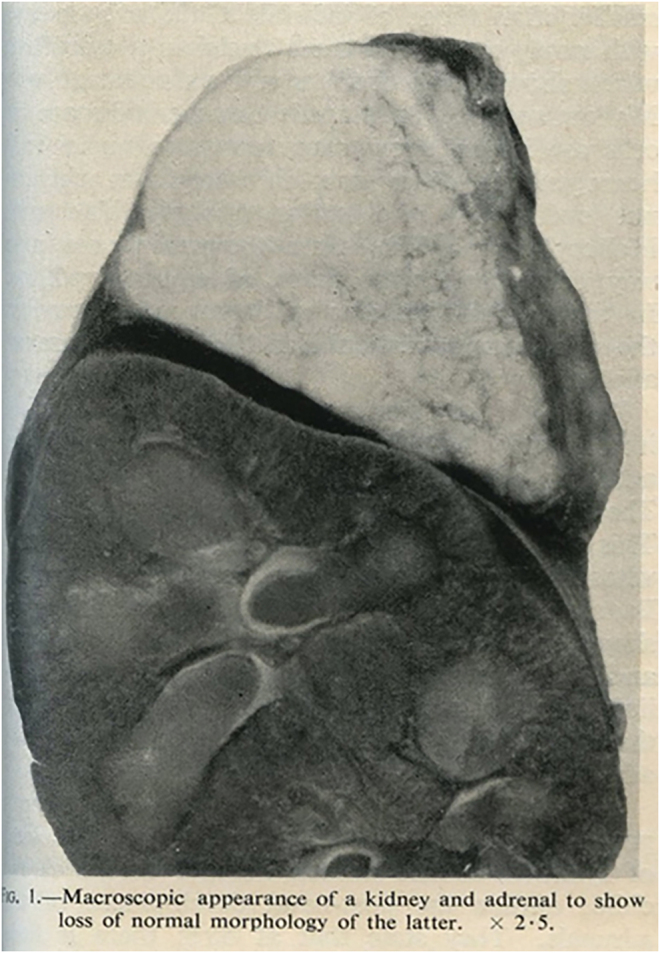
Sandison’s illustration of the sectioned kidney and adrenal from the patient he reported with ‘lipidosis of the adrenal’. From Sandison (1955), reproduced with permission.

In 1955, two reports appeared describing what is now called ‘lipoid congenital adrenal hyperplasia’ or ‘lipoid CAH’. Andrea Prader in Zurich described the autopsy of a 6-week-old phenotypic female who died in an Addisonian crisis and whose autopsy showed no uterus, tubes or ovaries, massive adrenals and no detectable nuclear Barr bodies (Prader & Gurtner 1955). Contemporaneously, AT Sandison in Glasgow reported the autopsy of a 3-month-old infant who also died in an apparent Addisonian crisis; she had a normal uterus, tubes and ovaries and no lipid deposits except in the massively enlarged adrenals (Sandison 1955) (Fig. 1). Neither Prader nor Sandison reported any steroid data. In 1957, Prader reported another case in a genetically female infant who was (initially) successfully treated with steroids; based on the large, lipid-laden adrenals seen at autopsy, he introduced the term ‘lipoid CAH’ and deduced that this was a genetic disorder of steroidogenesis but incorrectly suggested that the defect was in the conversion of Δ5 to Δ4 steroids (Prader & Siebenmann 1957). In 1961, Shimuzu *et al.* showed that cholesterol was converted to pregnenolone via 20αOH and 22-OH intermediates, suggesting the existence of three enzymes: a 20α-hydroxylase, a 22-hydroxylase and a 20,22-desmolase (Shimizu *et al.* 1961). The first clinical report of lipoid CAH in English appeared in 1964 and reviewed earlier cases (O’Doherty 1964). In 1968, Claude Migeon’s laboratory deduced that lipoid CAH was a disorder in the conversion of cholesterol to pregnenolone and suggested that the defect was in one of the steps described by Shimuzu (Camacho *et al.* 1968). Simpson and Boyd showed that the conversion of cholesterol to pregnenolone occurred in mitochondria and required a cytochrome P450 enzyme (now termed P450scc or CYP11A1) (Simpson & Boyd 1966, 1967). In 1974, Shikita and Hall showed that cholesterol was converted to 20α-hydroxycholesterol, then to 20,22-dehydrocholesterol and finally to pregnenolone with equimolar stoichiometry, in which each of the three reactions required one ‘TPNH’ (NADPH) (Shikita & Hall 1974). In 1972, Degenhart *et al.* reported that control adrenal mitochondria could convert cholesterol to pregnenolone, but that adrenal mitochondria from a patient with lipoid CAH could not; however, the patient’s mitochondria did convert 20α-hydroxycholesterol to pregnenolone, suggesting that a defect was in a specific 20α-hydroxylase (Degenhart *et al.* 1972). This logical conclusion turned out to be incorrect, because soluble hydroxysterols, such as 20α-hydroxycholesterol, can freely enter the mitochondria and be converted to pregnenolone (Toaff *et al.* 1982), without the help of the then-unknown disordered step in lipoid CAH, which turned out to be StAR (Lin *et al.* 1995). In 1977, Koizumi *et al.* reported that adrenal mitochondria from a patient with lipoid CAH had normal P450-mediated 11β-hydroxylase and 18-hydroxylase activities but could not convert cholesterol to pregnenolone; this suggested that the lipoid CAH mitochondria lacked the mitochondrial cholesterol side-chain cleavage enzyme, P450scc (Koizumi *et al.* 1977).

Taken together, these reports were regarded as showing that lipoid CAH was a defect in the enzymatic conversion of cholesterol to pregnenolone; hence, the disorder was clinically termed ‘20,22 desmolase deficiency’, i.e., a defect in P450scc; by 1985, 32 cases of lipoid CAH had been reported (Hauffa *et al.* 1985). When human P450scc was cloned (Chung *et al.* 1986), mutations in P450scc were sought in patient DNA (Matteson *et al.* 1986, Lin *et al.* 1991, Sakai *et al.* 1994, Fukami *et al.* 1995); mutations were also sought in its electron-donating cofactors (ferredoxin and ferredoxin reductase) and some factors (sterol carrier protein-2 (SCP2), endozepine, the 78-kDa glucose-regulated protein (GRP78) and peripheral benzodiazepine receptor (PBR)/TSPO) thought to facilitate the entry of cholesterol into mitochondria, but no mutations were found (Lin *et al.* 1991, Lin *et al.* 1993). Hence, lipoid CAH was not ‘20,22-desmolase deficiency’. Furthermore, placental synthesis of progesterone persisted in lipoid CAH pregnancies, permitting term gestation (Saenger *et al.* 1995).

Thus, the gene mutated in lipoid CAH was expressed in the adrenals and gonads, but not in placenta (or presumably in other steroidogenic tissues), but the responsible gene remained unknown.

## Acute vs chronic regulation of adrenal steroidogenesis

Endocrine cells that produce polypeptide hormones contain large amounts of their product(s) in secretory vesicles, a fact that was key to the purification of insulin, growth hormone, etc. However, steroidogenic cells lack such vesicles and contain very small amounts of the hormones they produce. An early study comparing steroid quantities extractable from adrenals, peripheral tissues and blood showed that the 24-h corticosteroid secretion of a 10-kg dog was comparable to the amount found in 17.3 kg adrenal tissue (Vogt 1943), suggesting that steroids were released almost as soon as they were produced, with little intraglandular storage. Thus, whereas the synthesis and release of polypeptide hormones are regulated separately, the synthesis and release of steroid hormones are constitutively linked, with minimal intracellular storage. As hormone-producing tissues are not readily accessible for clinical study, endocrinologists typically infer hormone production by measuring hormone concentrations in blood. Many years of work were required to show that while P450scc is the enzymatic rate-limiting step in steroidogenesis, the rate-limiting step in the immediate response to ACTH (or LH) is the access of cholesterol to P450scc (Stocco & Clark 1996, Miller & White 2023).

Ferguson (Ferguson 1963) and Garren (Garren *et al.* 1965, Davis & Garren 1968, Garren 1968) used inhibitors of protein synthesis (puromycin and cycloheximide) to show that the action of ACTH on the adrenal was not direct but required the synthesis of one or more additional protein(s). This action was rapid; the newly synthesized, cycloheximide-sensitive protein(s) had a short half-life and appeared to promote the delivery of cholesterol to P450scc on the inner mitochondrial membrane (IMM) (Simpson & Boyd 1966, Davis & Garren 1968, Privalle *et al.* 1983).

The P450scc system is the enzymatic rate-limiting step in steroidogenesis (Simpson 1979, Kimura 1981) and is among the slowest enzymes in nature, with a net turnover number of <20 molecules of cholesterol being converted to pregnenolone per molecule of P450scc per second (Tuckey & Cameron 1993). Because cholesterol is insoluble in water, other factors are needed for it to reach the mitochondria and initiate steroidogenesis; this constitutes the rate-limiting step in steroidogenesis (Crivello & Jefcoate 1980). By 1987, it was well established that the mitochondrial import of cholesterol, not the inherent enzymology of P450scc, was rate-limiting (Jefcoate *et al.* 1987, Lambeth *et al.* 1987). These and other studies led to the model that the acute regulation of steroidogenesis required a hormonally stimulated, rapidly synthesized, labile protein that appeared to mediate the transfer of cholesterol across the two mitochondrial membranes to reach P450scc (Hall 1985, Stocco & Clark 1996). Several candidates were proposed to function as this rapidly synthesized protein regulator of steroid biosynthesis, notably the ‘steroidogenesis-activator polypeptide’ (Pedersen & Brownie 1987), which is the carboxy-terminal 30 amino acids of GRP78 (Li *et al.* 1989), but essential roles for these proteins were eventually ruled out; these and other ‘dead-ends’ have been reviewed elsewhere (Stocco & Clark 1996, Miller & Bose 2011, Stocco *et al.* 2017).

**Figure 2:**
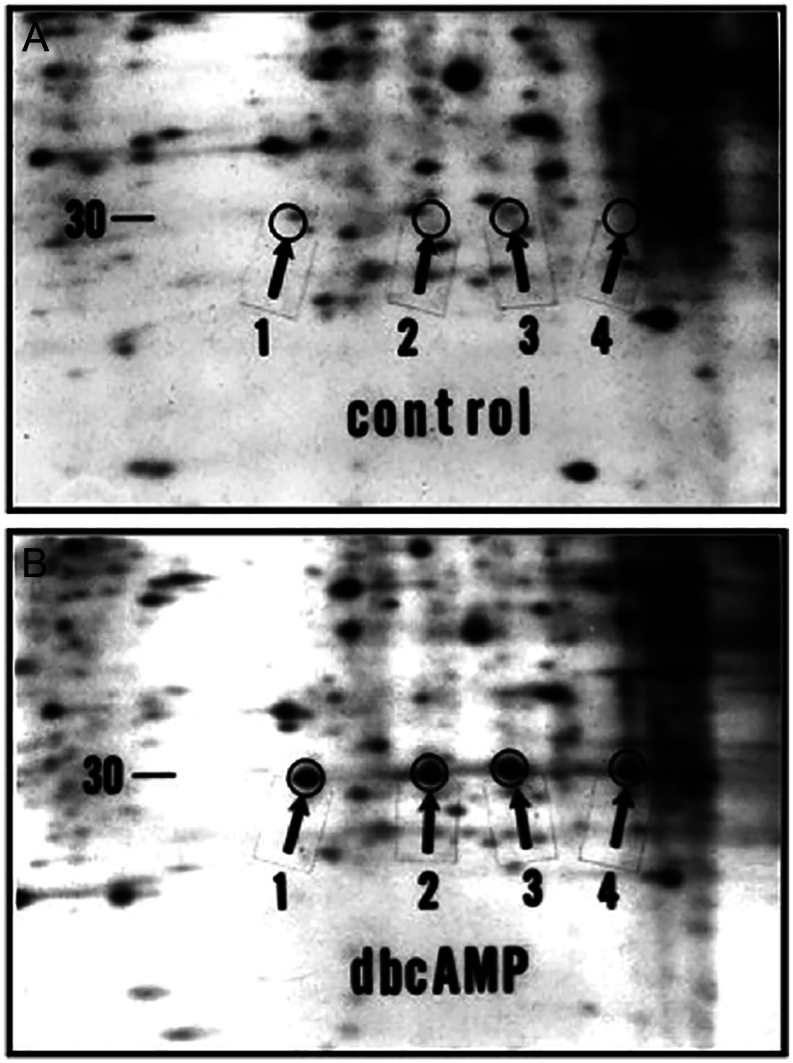
2D gels of mitochondrial proteins showing newly synthesized StAR in cultured mouse MA-10 Leydig cells treated without (panel A) or with (panel B) 1 mM dibutyryl cAMP (dbcAMP) for 6 h. Cells were incubated with ^35^S-methionine with or without 1 mM dbcAMP. The arrows illustrate the positions of the 30 kDa StAR protein forms. Isoforms 3 and 4 are the phosphorylated forms of isoforms 1 and 2, respectively. Figure courtesy of DM Stocco, from Stocco & Clark (1996), reproduced with permission.

The responsible protein was first identified by Nanette Orme-Johnson and colleagues at Tufts University using 2D gel electrophoresis of newly synthesized proteins labeled *in vitro* with radioactive phosphorus. A 30-kDa ACTH-induced mitochondrial phosphoprotein was found in mouse adrenocortical cells, and a similar LH-induced protein was found in rat corpus luteum cells and mouse MA-10 Leydig cells (Krueger & Orme-Johnson 1983, Pon & Orme-Johnson 1986, Pon *et al.* 1986, Alberta *et al.* 1989, Epstein & Orme-Johnson 1991); importantly, the appearance of this ‘spot’ on the 2D gels was temporally related to the induction of steroid hormone biosynthesis. Douglas M Stocco and colleagues at Texas Tech University Health Sciences Center identified a similar candidate mitochondrial protein in mouse MA-10 Leydig cells (Fig. 2) (Stocco & Chen 1991, Stocco & Sodeman 1991). Stocco and Clark extracted some of this protein from the gels, subjected it to microsequencing, used the partial amino acid sequence to design oligonucleotide probes, screened an MA-10 cell cDNA library, and isolated a candidate clone. That cDNA encoded an open reading frame for an unknown protein; when that cDNA was expressed in MA-10 cells, it induced steroid hormone synthesis even in the absence of LH stimulation (Clark *et al.* 1994).

This new factor was named the steroidogenic acute regulatory protein (StAR).

## Lipoid CAH. Nature’s StAR knockout

**Figure 3:**
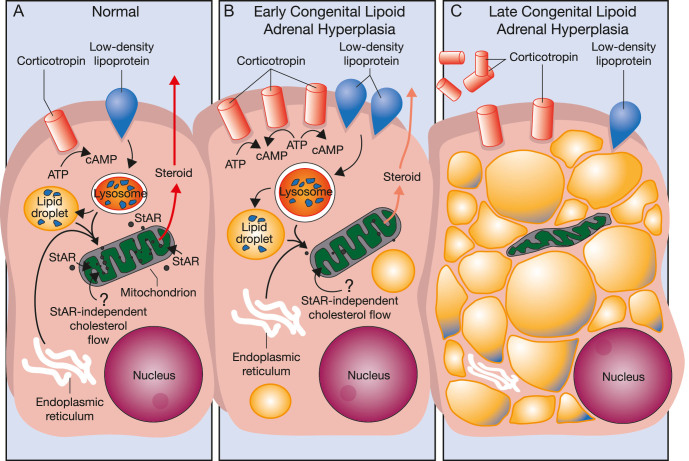
Two-hit model of lipoid CAH. (A) In normal human adrenal cells, cholesterol is primarily derived from low-density lipoproteins, with some endogenous synthesis in the endoplasmic reticulum. The rate-limiting step in steroidogenesis is the flow of cholesterol from the OMM to the IMM, which is facilitated by StAR. (B) Early in lipoid CAH, StAR-independent mechanisms continue to provide some cholesterol, permitting a low level of steroidogenesis. In response to low adrenal steroidogenesis, ACTH secretion increases, stimulating further accumulation of cholesterol and cholesterol esters in lipid droplets. (C) As lipid droplets accumulate, they engorge and damage the cell through physical displacement and by the action of cholesterol auto-oxidation products. The steroidogenic capacity is destroyed, but tropic stimulation continues. In the ovary, follicular cells remain unstimulated and undamaged until puberty when small amounts of estradiol are produced, as in panel B, causing partial feminization, with infertility and hypergonadotropic hypogonadism. Data Bose *et al.* (1996).

The cloning of StAR led to many questions and to a rapid explosion in research. Is StAR essential? What happens in its absence? How is it regulated? Does it act alone? And most importantly, how does it work? To address whether StAR is essential, Stocco sought to knockout the mouse StAR gene in collaboration with the late Keith L Parker, who had achieved renown with the discovery of the transcription factor ‘steroidogenic factor-1’ and the demonstration that its knockout prevented mouse adrenal development (Luo *et al.* 1994). However, the author of this review, who had been studying lipoid CAH (Matteson *et al.* 1986, Lin *et al.* 1991, Lin *et al.* 1993), hypothesized that lipoid CAH might be the ‘StAR knockout of nature’ and proposed a collaboration with Stocco and Jerome F Strauss III at The University of Pennsylvania. This team quickly showed that StAR is mutated in lipoid CAH and showed that coexpression of StAR with the P450scc system in non-steroidogenic cells induced steroidogenesis (Lin *et al.* 1995). They then cloned the human StAR cDNA and showed that StAR is expressed in the adrenals and gonads but not placenta (Sugawara *et al.* 1995*a*); then, they cloned the human StAR gene and showed that StAR also stimulated 27-hydroxylase activity, suggesting a role in bile acid synthesis (Sugawara *et al.* 1995*b*). This work proved that StAR is the factor responsible for lipoid CAH, thus proving that StAR is essential for adrenal and gonadal but not placental steroidogenesis, and fulfilled the criteria for the acute regulator of steroidogenesis (Stocco & Clark 1996). A puzzling clinical feature of lipoid CAH was the unexpected appearance of normally timed, but incomplete features of puberty in affected 46,XX patients (Tanae *et al.* 1988, 2000, Matsuo *et al.* 1994), whereas the 46,XY patients typically had no external genital development or puberty. By clinical and cell biological investigation, Miller and Strauss deduced the two-hit pathophysiology of this disorder that explained this clinical phenotype (Bose *et al.* 1996) (Fig. 3). Fetal adrenals and testes are steroidogenically active; hence, mutant StAR prevented steroidogenesis in those tissues, but ovaries were unaffected until puberty when StAR-independent steroidogenesis produced enough estradiol to ‘feminize’ affected females. This model was soon confirmed clinically (Bose *et al.* 1997, Fujieda *et al. *1997) and by Parker’s knockout mice (Caron *et al.* 1997). A milder, ‘non-classic’ form of lipoid CAH without phenotypic sex reversal in 46,XY patients was reported ten years later (Baker *et al.* 2006) and has now been confirmed in multiple reports (Sahakitrungruang *et al.* 2010, Altinkilic *et al.* 2024, Phadte *et al.* 2024), greatly expanding the clinical situations in which StAR mutations must be considered.

Thus, an obscure endocrine disease, lipoid CAH, identified StAR’s essential physiological roles.

## Regulation of StAR

The principal tropic regulators of steroidogenesis are ACTH, acting on adrenal cells, and LH, acting on gonadal cells; in both cases, the principal second messenger is cAMP, which induces protein kinase A (PKA). This axis induces transcription of the genes encoding steroidogenic enzymes, principally the *CYP11A1* gene encoding P450scc, which is the slowest steroidogenic enzyme and is rate-limiting; this induction of P450scc synthesis constitutes the ‘chronic regulation’ of steroidogenesis (Voutilainen *et al.* 1986). PKA also induces the transcription of the *STAR* gene encoding StAR but does so much more rapidly than its induction of the *CYP11A1* gene (Manna *et al.* 2002). The action of angiotensin II to induce StAR expression in the adrenal zona glomerulosa does not appear to be mediated by cAMP/PKA, but this area is not well understood. The transcriptional regulation of the *STAR* gene is very complex and has been reviewed in detail for this collection of papers (Viger *et al.* 2024). In addition, PKA also phosphorylates extant StAR, principally at Ser195 (S195; S194 in mouse), approximately doubling StAR activity; the PKA anchor protein AKAP121 recruits the type II PKA regulatory subunit (PKAR2A) to phosphorylate StAR, whereas the type I kinase drives StAR transcription (Arakane *et al.* 1997, Stocco *et al.* 2005) (a list of non-standard abbreviations is provided in Table 2). However, in StAR knockout mice, returning StAR to the steroidogenic cells (‘knockin’ of WT StAR) rescued embryonic lethality, but knockin of S194A StAR did not, suggesting that phosphorylation of Ser194/195 was essential (Sakai *et al.* 2014).

Thus, the chronic regulation of steroidogenesis is based on establishing the cell’s steroidogenic machinery, whereas the acute regulation is based on the delivery of the steroidogenic substrate, cholesterol, to that machinery.

**Table 2:** Abbreviations used.

ACAT	Acyl-coenzyme A:cholesterol acyltransferase
AKAP121	AKA anchoring protein 121
ANT	Adenine nucleotide transporter
CAH	Congenital adrenal hyperplasia
FDX	Ferredoxin
FDXR	Ferredoxin reductase
HSP60	Heat shock protein 60
IMM	Inner mitochondrial membrane
IMS	Intra-membranous space
MENTHO	MLN64 N-terminal homolog
MLN64	Metastatic lymph node clone 64
NPC	Niemann–Pick type C disease
OMM	Outer mitochondrial membrane
PAP7	PKA regulatory subunit RIα-associated protein 7
PBR	Peripheral benzodiazepine receptor
PKA	Protein kinase A
PKAR2A	PKA regulatory subunit 2
PRAX1	TSPO-associated protein-1
START	StAR-related lipid transfer
Tom	Translocase of outer membrane
TSPO	Translocator protein
VDAC	Voltage-dependent anion channel

## Where does StAR act?

The mechanism of StAR’s action has been studied extensively but remains incompletely understood (Miller 2007, Miller & Bose 2011, Miller 2017*a*,*b*). In the absence of data about StAR’s three-dimensional structure, initial studies focused on cell biological and biophysical studies. The 2D gel studies of Orme-Johnson and Stocco (see the ‘Acute vs chronic regulation of adrenal steroidogenesis’ section above) identified two major forms of StAR: a 37-kDa cytoplasmic form with a short half-life of ∼15 min and a much more abundant 30-kDa intramitochondrial form with a half-life of several hours; each of these could be identified in both phosphorylated and non-phosphorylated forms, in both adrenal cells and testicular Leydig cells. The amino acid sequences of mouse (Clark *et al.* 1994) and human (Sugawara *et al.* 1995*a*) StAR, as revealed by cDNA cloning, included arginine-rich amino termini – classical ‘mitochondrial leader sequences’. By analogy with known mitochondrially targeted proteins that act within the mitochondrion, 37-kDa StAR was quickly termed the ‘precursor’ form (retaining the mitochondrial leader sequence) and 30-kDa StAR was termed the ‘mature’ form, lacking the leader. This classical arrangement was quickly questioned because deleting up to 62 amino-terminal residues (N-62 StAR), including the mitochondrial leader, confined StAR to the cytoplasm but had no effect on StAR’s activity in transfected cells, and immunogold electron microscopy showed the N-62 StAR associated with the outer mitochondrial membrane (OMM), even though it lacked a mitochondrial-targeting sequence (Arakane *et al.* 1996).

Thus, StAR acts prior to its importation into mitochondria.

## StAR as a molten globule

Until the discovery that StAR mutations cause lipoid CAH (Lin *et al.* 1995), all then-known forms of CAH were caused by mutations in enzymes, most of which were forms of cytochrome P450. By that time, massive amounts of work had been done characterizing drug-metabolizing P450s (Wrighton & Stevens 1992) and several bacterial P450 enzymes had been crystallized (Li & Poulos 1994); thus, it was possible to infer the structural and enzymatic consequences of mutations causing various forms of CAH. Many different StAR mutations could cause lipoid CAH (Bose *et al.* 1996, Nakae *et al.* 1997), but it was unclear how these mutations disrupted a structure or function that had not yet been established. In the absence of a crystallographic structure for StAR, early work examined bacterially expressed StAR mutants by biophysical approaches. Circular dichroism, UV spectroscopy and Fourier-transform infrared spectroscopy showed that StAR mutants causing severe lipoid CAH were misfolded, while the folding of mutants that retained partial activity resembled wild-type StAR (Bose *et al.* 1998). This work led to more complex analyses demonstrating that wild-type StAR undergoes a pH-dependent change in its folding, termed as a molten globule transition (Bose *et al.* 1999).

Most proteins are folded into unique conformations determined by energetic information specified by their amino acid sequences; folding typically proceeds through intermediate states with decreasing free energies. Partially folded intermediates that have substantial secondary structure but have not achieved their final tertiary structure may be termed ‘molten globules’, which are dynamic proteins that retain substantial α-helical structure but lack some tertiary structure (Ptitsyn 1995, Privalov 1996, Gupta & Uversky 2023). Molten globules may be ‘wet’ or ‘dry’ depending on the degree to which water has entered the otherwise hydrophobic protein core (Jha & Udgaonkar 2009); most of them, including StAR, are wet. As incompletely folded structural intermediates, molten globules generally lack biological activity, although membrane-bound proteins may go through molten globule states during membrane insertion (Van der Goot 1991). At acidic pH, StAR behaves as a molten globule (Bose *et al.* 1999). While the cytoplasm is not acidic, the study suggested that StAR’s interaction with protonated phospholipid head groups on the OMM may provide the proton-rich environment needed for a molten globule transition. This was then demonstrated *in vitro* with synthetic dansylated phospholipid vesicles assessed by fluorescence energy transfer (Christensen *et al.* 2001) and *in silico* by molecular dynamic modeling (Baker *et al.* 2005, Murcia *et al.* 2006). From the perspective of StAR’s cell biology, the surprising discovery of StAR’s molten globule behavior is wholly logical. StAR acts outside the mitochondria, where it must interact with and deliver large amounts of cholesterol into the mitochondria; StAR’s versatile folding state makes this possible, whereas the rigid protein seen by crystallography would not. As discussed below, other StAR-like proteins also undergo molten globule transitions.

Thus, StAR is active as a molten globule, while it is associated with the OMM.

## StAR-independent steroidogenesis

As discussed in the ‘Lipoid CAH’ section above, lipoid CAH was initially thought to be a defect in P450scc, but no defects were found in P450scc, ferredoxin (FDX), ferredoxin reductase (FDXR) or in some factors thought to facilitate cholesterol’s entry into the mitochondria. Furthermore, placental steroidogenesis in lipoid CAH pregnancies was unaffected. The placenta is a fetal tissue that expresses P450scc (Chung *et al.* 1986), and placentally produced progesterone is needed to suppress uterine contractility, permitting term gestation, but the placenta does not mount an acute steroidogenic response. Thus, the gene that is mutated in lipoid CAH had to be expressed in the adrenals and gonads, but not in placenta. This deduction was key in identifying StAR’s role in lipoid CAH and also stimulated exploration of how the placenta can produce abundant steroids in the absence of StAR. Non-steroidogenic cells transfected with the side-chain cleavage system (P450scc/FDX/FDXR) synthesize pregnenolone in the absence of StAR at about 14% of the StAR-induced rate (Lin *et al.* 1995); this observation led to the formulation of the ‘two-hit model’ of lipoid CAH (Bose *et al.* 1996). Similar StAR-independent steroidogenesis is seen in brain, skin and other tissues. Two mechanisms may account for StAR-independent steroidogenesis. First, soluble hydroxysterols are freely diffusible into the mitochondria and are effective substrates for P450scc (Toaff *et al.* 1982); this circumvention of StAR’s action underlies the use of hydroxysterols *in vitro* to determine the net steroidogenic capacity of cells carrying P450scc and could account for low levels of steroidogenesis in cells lacking StAR. Second, other proteins might substitute for StAR. It is also possible that both tactics are used in the placenta.

StAR facilitates the flow of cholesterol from the OMM to the IMM. The suggestion that StAR delivers cholesterol from cellular stores to the OMM is stoichiometrically improbable. Crystallography indicates that each molecule of StAR binds only one molecule of cholesterol (Tsujishita & Hurley 2000) and StAR’s mitochondrial leader affixes it to the OMM; hence, this is a one-way trip: once a StAR molecule gets to a mitochondrion, it is stuck there and cannot go back to pick up another molecule of cholesterol (Bose *et al.* 2002). Nevertheless, each StAR molecule delivers hundreds of molecules of cholesterol from the OMM to the IMM (Artemenko *et al.* 2001). Thus, other mechanisms must be invoked to explain the delivery of large amounts of cholesterol to the OMM. There are no known genetic disorders of this process, and mouse knockouts of candidate factors have yielded minimal phenotypes, suggesting that multiple mechanisms are involved in this process, providing functional redundancy. Substantial attention has been directed to proteins structurally related to StAR, including MLN64, and to the StarD4/StarD5/StarD6 proteins.

Nevertheless, the movement of cholesterol to the OMM remains under investigation.

## MLN64

The placenta is one of many tissues that express MLN64 (metastatic lymph node clone 64), a 445-amino acid protein cloned from metastatic breast carcinoma cells (Moog-Lutz *et al.* 1997). The carboxy-terminal 227 amino acids of MLN64 are 37% identical and 50% similar to the sequence of StAR. Although full-length 445-amino acid MLN64 lacks StAR-like activity, its isolated carboxy-terminal domain lacking 234 amino-terminal residues (N-234 MLN64) has about half of StAR’s activity to promote steroidogenesis in transfected cells (Watari *et al.* 1997). A similarly truncated form of MLN64 is expressed in human placenta (Bose *et al.* 2000*b*, Tuckey *et al.* 2004, Olvera-Sanchez *et al.* 2011). Although this form of MLN64 has not been rigorously characterized at the molecular level, this protein may interact with a cytoplasmic heat shock protein (HSP60) to stimulate steroidogenesis in placental mitochondria (Olvera-Sanchez *et al.* 2011). Spectroscopic studies and partial proteolysis of bacterially expressed N-62 StAR (Bose *et al.* 1999) and N-234 MLN64 (Bose *et al.* 2000*b*) show that both proteins undergo pH-dependent molten globule transitions, suggesting that such a structural transition would occur when StAR interacts with the protonated phospholipid head groups on the OMM. However, decreasing expression of MLN64 *in vitro* by 90% only decreased progesterone synthesis by 30% (Charman *et al.* 2010).

MLN64 has a partner protein termed MENTHO (MLN64 N-terminal homolog, also termed StarD3NL); both are late endosomal proteins that co-localize with the NPC proteins associated with Niemann–Pick type C disease (Zhang *et al.* 2002). MENTHO has 70% identity, 83% similarity and a gene structure similar to the N-terminal domain of MLN64 (Alpy *et al.* 2002, Alpy *et al.* 2005). MLN64 and MENTHO may act downstream from the NPC proteins in the trafficking of cholesterol in peroxisomes and lipid droplets, but disruption of the cholesterol-binding domain of MLN64 in transgenic mice yields no obvious phenotype, as the mice are fertile and neurologically intact (Kishida *et al.* 2004). Thus, MLN64 is not solely responsible for cholesterol entry into placental syncytiotrophoblast cells; if MLN64 plays a role in cholesterol trafficking, it appears that other factors can compensate for its absence.

Thus, other proteins can exert StAR-like activity that may contribute to StAR-independent steroidogenesis.

## START domain proteins

Because it is generally assumed that ‘structure dictates function’, there was great interest in determining StAR’s three-dimensional structure. Bacterially expressed StAR tended to aggregate *in vitro*, frustrating early efforts at crystallization or to determine its structure by nuclear magnetic resonance spectroscopy (Song *et al.* 2001). In 1999, computational searches of known amino acid sequences revealed that StAR was the foundational member of a family of proteins involved in lipid binding or transfer (Ponting & Aravind 1999). These proteins are found throughout eukaryotes, especially in plants; the mammalian branch of this family comprises 15 members that have similar sequences in a ‘StAR-related lipid transfer (START)’ domain, a conserved sequence of ∼210 amino acids that binds sterols and other lipids (Iyer *et al.* 2001). The mammalian START domain proteins are termed StarD1 through StarD15 (Soccio & Breslow 2003, Alpy & Tomasetto 2014, Clark 2020). In this nomenclature, StAR is StarD1 and MLN64 is StarD3. The closely related StarD4, StarD5 and StarD6 proteins appear to be cytosolic proteins involved in non-vesicular transport of sterols (Soccio *et al.* 2002).

The START domain of MLN64 (N-216 MLN64) was the first StAR-like molecule whose structure was determined by crystallography, revealing a globular protein with an α/β helix-grip fold and a nine-stranded anti-parallel β-sheet forming an elongated, U-shaped hydrophobic pocket that can accommodate a molecule of cholesterol (Tsujishita & Hurley 2000). Crystallography and computational modeling eventually showed that all START domain proteins have very similar structures (Thorsell *et al.* 2011). Partial proteolysis of StAR interacting with artificial membranes indicated that only very limited portions of the StAR molecule interact with the OMM (Yaworsky *et al.* 2005). Molecular dynamics simulations, cysteine cross-linking, mutagenesis studies and direct biophysical measurements confirmed complex, pH-dependent molten globule transitions when StAR interacts with the OMM (Baker *et al.* 2005, Rajapaksha *et al.* 2013, Iaea *et al.* 2015).

The sequences of StarD4, StarD5 and StarD6 are closely related to StAR but lack leader peptides that would target them to specific organelles, suggesting that they may play roles in transcytoplasmic cholesterol transport (Soccio & Breslow 2003). StarD4 and StarD5 have low levels of StAR-like activity in transfected cells (Bose *et al.* 2008*b*), but these proteins are mainly expressed in liver and kidney, and knockout of the StarD4 gene in mice does not disrupt steroidogenesis (Riegelhaupt *et al.* 2010). StarD4 is found in close proximity to acyl-coenzyme A:cholesterol acyltransferase (ACAT) in the ER and appears to stimulate ACAT activity (Rodriguez-Agudo *et al.* 2011). StarD5 can shuttle cholesterol from the plasma membrane to the ER in renal tubule cells (Chen *et al.* 2009) but is probably more important in the intracellular movement of bile acids (Létourneau *et al.* 2012). Thus, StarD4 and StarD5 may be important for intracellular cholesterol shuttling but probably are not crucially involved in steroidogenesis. StarD6 is expressed in male germ line cells and in the (porcine) ovarian corpus luteum (LaVoie *et al.* 2014) and has greater StAR-like activity than StAR itself in assays *in vitro* (Bose *et al.* 2008*b*), but its pattern of expression is inconsistent with a general role in steroidogenesis.

Thus, the mechanisms by which steroidogenic cells move large amounts of cholesterol from various cellular depots to the IMM for subsequent action by StAR remain under investigation.

## Implications of StAR’s structure

StAR has challenged the dictum of protein chemistry that ‘structure dictates function’. The crystal structure of N-216 MLN64 was interpreted to indicate that StAR functioned as an intramolecular cholesterol shuttle, presumably located in the mitochondrial intramembranous space (IMS), taking cholesterol from the OMM and delivering it to the IMM (Tsujishita & Hurley 2000). However, in 2002, Bose, Lingappa and Miller showed that: i) the 37- and 30-kDa forms of StAR are equally active when expressed in the cytoplasm or when added to isolated steroidogenic mitochondria *in vitro*; ii) StAR is constitutively active when confined to the cytoplasmic aspect of the OMM but inactive when localized to the IMS or to the matrix side of the IMM; and iii) constructs that slow StAR’s mitochondrial import and increase its residency time on the OMM increase its activity, whereas constructs that speed up its mitochondrial entry decrease its activity (Bose *et al.* 2002). That study showed that StAR’s mitochondrial leader not only targets it to the mitochondrion but also affixes it there. Thus, StAR makes a one-way trip to the OMM; StAR might transport one molecule of cholesterol into the mitochondrion, but after StAR enters the mitochondrion, it cannot exit and repeat such a cycle. This highlights the stoichiometric problem of cholesterol import: how much cholesterol can one StAR molecule deliver to P450scc on the IMM? In experiments examining cholesterol transfer between synthetic phospholipid vesicles, each molecule of StAR could transfer about 2.8 molecules of cholesterol (Tuckey *et al.* 2002), but in experiments *in vivo*, each molecule of StAR transferred hundreds of molecules of cholesterol into mitochondria (Artemenko *et al.* 2001).

Thus, StAR acts exclusively on the OMM; its activity is proportional to its residency time on the OMM, and it is StAR’s cellular localization, and not its cleavage from 37 to 30 kDa, that determines its activity.

## The proposed role of the peripheral benzodiazepine receptor (PBR)

Cholesterol is essentially insoluble in water (or cytoplasm); hence, it requires some form of assistance to reach and enter steroidogenic mitochondria; this ‘assistance’ can be in the form of vesicular or non-vesicular trafficking (Miller & Bose 2011). By 1980, it was clear that the delivery of cholesterol to P450scc, rather than the enzymatic activity of P450scc, was the biologically rate-limiting step in steroidogenesis, even though P450scc was the enzymatically rate-limiting step (Crivello & Jefcoate 1980). Many proteins were proposed to act in this process (non-vesicular trafficking); in the 1980s, it was widely thought that cholesterol was transported to mitochondria by cytoplasmic SCP2 (Chanderbhan *et al.* 1982, Vahouny *et al.* 1983) and that mitochondrial entry was facilitated by ‘steroidogenesis-activator peptide’ (Pedersen & Brownie 1987) derived from glucose regulatory protein GRP78 (Li *et al.* 1989). Soon thereafter, three laboratories reported that ligands of the PBR translocate cholesterol into mitochondria (Besman *et al.* 1989, Yanagibashi *et al.* 1989, Krueger & Papadopoulos 1992). Another laboratory associated PBR with the voltage-dependent anion channel (VDAC1) on the OMM and the adenine nucleotide carrier (ANT) on the IMM, the first description of a PBR receptor complex (McEnery *et al.* 1992). Thus began an extended, >30-year, controversy about the steroidogenic role, or lack of a role, for PBR (now renamed ‘translocator protein’ or TSPO).

Although the tissue distribution of PBR/TSPO and the kinetics of its response to cAMP did not seem to indicate a factor mediating the acute steroidogenic response (Stocco & Clark 1996), several studies indicated that PBR/TSPO acted downstream from StAR (Papadopoulos 1993, Hauet *et al.* 2005, Liu *et al.* 2006). Other proteins were reported to interact with PBR/TSPO, forming a multimolecular machine dubbed the ‘transduceosome’, consisting of PBR/TSPO itself, the 34-kDa VDAC1, the 30-kDa ANT, a 10-kDa protein (pk 10), the TSPO-associated protein-1 (PRAX-1) and the TSPO and protein kinase A (PKA) regulatory subunit RIα-associated protein 7 (PAP7, also known as acyl-CoA-binding domain containing protein 3 or ACBD3) (Rone *et al.* 2009). The proposed role of PBR/TSPO in steroidogenesis was supported by four lines of evidence: i) pharmacologic experiments *in vitro*, principally based on the synthetic TSPO ligand PK11195 (Papadopoulos 1993); ii) modeling, cross-linking and drug-binding studies indicating that PBR/TSPO has a cytoplasmic domain containing a ‘cholesterol-recognition amino acid consensus’ (‘CRAC’) domain (Li *et al.* 2001); iii) reduced steroidogenesis when PBR/TSPO was knocked down in cells expressing a StAR construct affixed to the OMM (Hauet *et al.* 2005); and iv) most importantly, a review paper stating that knockout of PBR/TSPO in mice caused embryonic lethality, although no data supporting this conclusion were shown (Lacapere & Papadopoulos 2003). However, in 2014, two laboratories reported that steroidogenesis is unaffected in testis-specific (Morohaku *et al.* 2014) and global (Banati *et al.* 2014, Tu *et al.* 2014) knockouts of mouse PBR/TSPO; in 2016, a third laboratory reported normal viability in PBR/TSPO knockout mice (Wang *et al.* 2016). These results cast serious doubt on the proposed role of this factor in steroidogenesis (Gut *et al.* 2015, Selvaraj & Stocco 2015). Several follow-up papers appeared, supporting a steroidogenic role for PBR/TSPO, notably report of another mouse knockout (Fan *et al.* 2020); however, it has been suggested that both sexes of *Cre* mice may have been used in that study, confounding the results (Selvaraj *et al.* 2020). Many other tissue-specific and global mouse and rat PBR/TSPO knockouts have been reported; most find little or no impact on basal steroidogenesis, although some differences in steroidogenic responses to stress have been reported (Brehat *et al.* 2024). In addition, hamsters do not express PBR/TSPO in their adrenals, yet make steroids like other mammals (Koganti & Selvaraj 2020).

Thus, while PBR/TSPO may play a role in steroidogenesis in some tissues in some species, it is clear that PBR/TSPO does not play an essential, indispensable role in adrenal or gonadal steroidogenesis.

## Importation of cholesterol and StAR itself

It is now clear that StAR’s action to promote the acute steroidogenic response happens on the OMM and that its mitochondrial import terminates this action (Bose *et al.* 2002, Miller & Bose 2011). Much work was done from 2002 to 2024 addressing StAR’s importation into mitochondria. Studies of StAR’s N-terminal sequence, beginning with the N-terminal deletion mutagenesis studies (Arakane *et al.* 1996), have shown that amino acids 1–30 constitute the ‘mitochondrial leader’ sequence that targets StAR to the OMM and amino acids 31–62 constitute a ‘pause transfer’ sequence, which slows StAR’s mitochondrial entry, permitting longer occupancy time on the OMM, thus increasing its activity (Bose *et al.* 2002, Prasad *et al.* 2015, Bose *et al.* 2023). Exchanging the mitochondrial leader peptides of StAR, P450scc and other mitochondrial proteins showed that StAR’s slow mitochondrial importation is unusual and possibly unique, complicating its study (Rajapaksha *et al.* 2013). The initial cloning of StAR led to the short-lived hypothesis that StAR brings the OMM and IMM into contact, permitting cholesterol to flow down a concentration gradient from the cholesterol-rich OMM to the cholesterol-poor IMM (Clark *et al.* 1994). The formation of steroidogenic OMM/IMM contact sites may be involved in steroidogenesis and involve the 67-kDa protein ATAD3, which is enriched at mitochondrial contact sites, is anchored to the IMM and appears to facilitate contact between the IMM and the OMM (Da Cruz *et al.* 2003, Gilquin *et al.* 2010).

How, then, does StAR facilitate the rapid import of a gross molar excess of cholesterol? The answer remains in development but may follow the following outline. Biophysical studies *in vitro* and *in silico* show that StAR binds cholesterol (Kallen *et al.* 1998, Tuckey *et al.* 2002, Tuckey *et al.* 2004, Baker *et al.* 2005, Murcia *et al.* 2006, Baker *et al.* 2007), but the crystal structures of MLN64 (Tsujishita & Hurley 2000), StARD4 (Romanowski *et al.* 2002) and phosphatidylcholine transfer protein (Roderick *et al.* 2002) show that there is not enough room for cholesterol to enter the sterol-binding pocket. Therefore, a conformational change is necessary for cholesterol binding. Cholesterol binding affects StAR’s conformation and flexibility; this molten globule configuration permits binding of multiple molecules of cholesterol and permits a disparity in the amount of cholesterol bound and the amount of cholesterol delivered into the mitochondria (Rajapaksha *et al.* 2013).

The source of cholesterol is probably the ER, rather than cholesterol pre-existing in the OMM. Although the movement of cholesterol has not been measured directly, it would seem that the amount of cholesterol available in the OMM is probably insufficient for physiological demands during an acute steroidogenic response; furthermore, removing large amounts of cholesterol from the OMM would likely distort mitochondrial structure and function. The interactions between the ER and mitochondria occur at the ‘mitochondria-associated membrane’ (MAM), a region of the ER that is rich in cholesterol (resembling a ‘cholesterol raft’) (Vance 1990, Csordas *et al.* 2006, Kornmann *et al.* 2009, Vance 2014). ‘Tethering proteins’ associate the MAM with the OMM. In the MAM, StAR interacts with the σ-1 receptor, which acts to tether the MAM to the OMM (Hayashi & Su 2007), facilitating interaction with VDAC2 (Marriott *et al.* 2012). The N-terminus of ATAD3 contains 50 amino acids that may insert into the ER, also acting as a tethering protein (Issop *et al.* 2015). At the MAM, GRP78 facilitates the proper folding of StAR (Prasad *et al.* 2017), which then interacts with VDAC1 (Bose *et al.* 2008*a*) and VDAC2 (Prasad *et al.* 2015) at the MAM–mitochondria junction, prior to StAR import. Partially unfolded StAR (the molten globule form) exerts maximal activity before being imported (Bose *et al.* 1999, Bose *et al.* 2002). Thus, the physical association of StAR with the MAM strongly suggests that the MAM is the source of steroidogenic cholesterol.

**Figure 4:**
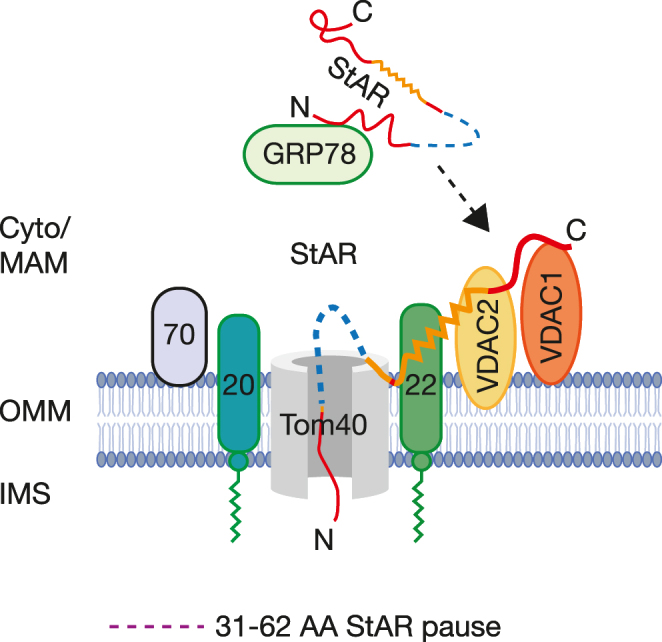
Current model of StAR’s importation into mitochondria; note that the IMM is not shown. Correct protein folding of StAR is facilitated by GRP78 at the MAM. StAR’s mitochondrial leader sequence (amino acids 1–30) then targets it to the mitochondrial protein import machinery on the OMM, while carboxy-terminal domains of StAR interact with VDAC1, VDAC2 and Tom22. StAR’s ‘pause sequence’ (amino acids 31–62) interacts with the Tom40 import channel, slowing its mitochondrial entry and permitting conformational changes (molten globule transition) needed to permit its activity before passing through the import channel. The means by which this complex facilitates cholesterol influx remains unknown. Figure courtesy of HS Bose; data Bose *et al.* (2023).

After facilitating cholesterol entry, the StAR protein is imported into mitochondria via the Tom (translocase of outer membrane) complex; Tom40 is the import channel that traverses the OMM (Bose *et al.* 2023) (Fig. 4). Other components of the mitochondrial protein import machinery, including Tom22 on the OMM and Tim50 on the IMM, apparently also participate in the mitochondrial importation of StAR (Bose *et al.* 2019, Bose *et al.* 2021). The mitochondrial importation of StAR is slow but eventually kills its action.

Thus, while our understanding of the mitochondrial importation of the StAR protein has advanced substantially, and more is now known about StAR’s interactions with cholesterol, it is still not clear how StAR facilitates the rapid, massive influx of cholesterol that is needed for the acute steroidogenic response.

## The intramitochondrial fate of StAR

As discussed above, the action of StAR on the OMM requires a pH-dependent molten globule transition (Bose *et al.* 1999, Baker *et al.* 2005), but StAR is inactive within the mitochondria, where it is degraded by Lon protease (Granot *et al.* 2003, Granot *et al.* 2007, Bahat *et al.* 2014). The foundational, 2D gel, pulse-chase labeling experiments of Orme-Johnson and Stocco indicated that extramitochondrial 37-kDa StAR has a half-life of <5 min, whereas the intramitochondrial 30-kDa StAR has a half-life of 1–4 h (Epstein & Orme-Johnson 1991, Stocco & Sodeman 1991). This intramitochondrial 30-kDa StAR does not stimulate steroidogenesis and thus has no established function, but its long half-life raises the possibility of other action(s). StAR is expressed in the brain, which is steroidogenic, but lacks any known acute steroidogenic responses. Studies have found StAR in the stressed mouse heart (Casal *et al.* 2003, Ohtani *et al.* 2009, Anuka *et al.* 2013) and in the rat heart stressed by chronic infusion of angiotensin II (Bose *et al.* 2021), but those tissues lacked detectable P450scc; hence, the detected StAR was not apparently stimulating steroidogenesis in the same fashion as it does in the adrenals and gonads. That study found 30-kDa StAR in a 290-kDa complex on the IMM that also contained P450c11AS (aldosterone synthase, CYP11B2) and the mitochondrial translocase receptor, Tom22. As the 30-kDa StAR appeared to be required for aldosterone synthesis, that study hypothesized that the 30-kDa StAR facilitated the interaction of P450c11AS with Tom22 (Bose *et al.* 2021).

Nevertheless, no function for the 30-kDa StAR has been established; this remains an under-investigated question.

## Lipoid CAH phenocopies: P450scc deficiency

Discovering that lipoid CAH was due to StAR mutations did not disprove that a similar syndrome might be caused by defects in P450scc or its electron transfer proteins; in fact, both of these disease mechanisms have been described. Lipoid CAH fetuses reach term normally, and term gestation requires placentally produced progesterone (to suppress uterine contractility), indicating that the P450scc system functioned normally in the placentas of lipoid CAH fetuses (Lin *et al.* 1991). This suggested that no one could reach term gestation with P450scc deficiency (Miller 1998). However, clinical reality superseded that logic with the report of a patient with hormonal features of lipoid CAH, normal StAR genes on both alleles and a heterozygous P450scc mutation (Tajima *et al.* 2001). Further reports of homozygous P450scc mutations soon followed (Katsumata *et al.* 2002, Hiort *et al.* 2005, al Kandari *et al.* 2006, Kim *et al.* 2008); most of these patients had severe loss-of-function mutations and presented with severe, early-onset adrenal failure and complete phenotypic 46,XY sex reversal in genetic males (Kim *et al.* 2008), but the manifesting heterozygosity of the initially reported patient remains unexplained (Miller 2017*a*,*b*). At least 70 patients with P450scc mutations have been described (Phadte *et al.* 2023, Alhamoudi *et al.* 2024), including a milder, ‘non-classic’ form of P450scc deficiency with late-onset adrenal insufficiency and normal 46,XY male genital development, similar to non-classic lipoid CAH (Rubtsov *et al.* 2009, Sahakitrungruang *et al.* 2011). Many reported patients have been from Eastern Turkey and were homozygous for the missense mutation R451W (Guran *et al.* 2016).

The clinical and hormonal findings in patients with lipoid CAH due to StAR mutations and in patients with P450scc mutations are almost indistinguishable. Nevertheless, despite the clinical similarities in these two mechanistically distinct diseases, P450scc deficiency should not be considered a form of lipoid CAH, as the massively enlarged adrenals that give lipoid CAH its name are not seen in P450scc deficiency. Nevertheless, adrenal size alone cannot distinguish lipoid CAH from P450scc deficiency, as small adrenals have been reported in at least one mutation-proven case of classic lipoid CAH (Bose *et al.* 2000*a*). As neither the clinical/hormonal findings nor adrenal size can definitively discriminate between deficiencies of P450scc and StAR, DNA sequencing should be done in all suspected cases (Gucev *et al.* 2013). The clinical aspects and differential diagnosis of these (and other) steroidogenic deficiencies have recently been reviewed (Miller 2018).

The discovery of patients with P450scc mutations prompts the question of whether some patients may have defects in ferredoxin (FDX) and ferredoxin reductase (FDXR), the electron-transfer factors required for P450scc activity. These proteins also participate in the biosynthesis of Fe–S centers, which are used by many proteins. Vertebrates have two ferredoxins (FDX1 and FDX2); FDX1 is principally involved with steroidogenesis, while FDX2 is principally involved in the synthesis of Fe–S clusters (Sheftel *et al.* 2010, Rouault 2015). Substantial work has been done studying the roles of these proteins (Miller *et al.* 2025). As of late 2024, no mutations have been described in FDX1. Mutations in human FDXR were reported in 2017, causing a mitochondriopathy variably manifesting with optic atrophy, retinal dystrophy, neuropathic hearing loss, developmental delay and movement disorders (Paul *et al.* 2017, Peng *et al.* 2017); at least 77 such patients were reported from 2017 to 2023 (Miller *et al.* 2025). An unbiased sequencing study of 2,186 patients with optic neuropathy found five patients with FDXR mutations, all of whom also had neuropathic deafness (Rocatcher *et al.* 2023). Defects in adrenal steroidogenesis were to be expected (Miller 2017*b*, Miller 2021) but were not sought (and found) until very recently (Pignatti *et al.* 2024); the adrenal insufficiency in these patients can now be recognized and treated.

Thus, the study of StAR and lipoid CAH continues to influence our understanding of adrenal insufficiency.

## Conclusions

A wealth of data demonstrate that StAR is the long-sought trigger to the acute steroidogenic response of the adrenals and gonads. This conclusion is most powerfully established by the observation that StAR is the factor that is mutated in lipoid congenital adrenal hyperplasia, illustrating how scientific theories and models are most convincing when wholly independent approaches lead to the same conclusion. Following the discovery of StAR and the demonstration of its essential role, the next major question was how StAR achieved its biological activity. Much effort was directed toward establishing the structure of StAR, but understanding that structure has shed surprisingly little light on the question of how StAR works. Less direct, more complex studies have identified multiple potential proteins that may be interacting partners with StAR, and such studies now appear to show how StAR enters a mitochondrion, but while much has been learned about StAR’s mitochondrial entry, the precise molecular itinerary of a cholesterol molecule’s travels from cytoplasmic stores to P450scc on the IMM remains unknown. Future studies are needed to address at least four major questions. i) What is the true molar efficiency of StAR, and how are numbers >1 molecule of cholesterol per molecule of StAR achieved? ii) Is cholesterol binding needed for StAR’s activity? Two studies have addressed this question directly, yielding decidedly different answers (Baker *et al.* 2007, Roostaee *et al.* 2008). iii) A large literature suggests a role for PBR/TSPO in mitochondrial cholesterol entry, but recent transgenic mouse experiments falsify this proposition (at least in mice). Does PBR/TSPO play a less dramatic but still functionally important permissive role not revealed in knockout mouse studies, and if such a role is hypothesized, how could it be demonstrated convincingly? iv) The long half-life of intramitochondrial 30-kDa StAR (compared to the steroidogenically active extramitochondrial form(s)) suggests that this protein may have an as-yet unidentified activity. Is this true, and what experiments might reveal such an activity?

In conclusion, we are far from understanding everything we need to know about this fascinating protein.

## Declaration of interest

The author declares that there is no conflict of interest that could be perceived as prejudicing the impartiality of this work.

## Funding

The preparation of this review did not receive any specific grant from any funding agency in the public, commercial or not-for-profit sector.

## Author contribution statement

WLM researched the topic and wrote the paper.
